# The Impact of Speed of Play in Gambling on Psychological and Behavioural Factors: A Critical Review

**DOI:** 10.1007/s10899-017-9701-7

**Published:** 2017-06-22

**Authors:** Andrew Harris, Mark D. Griffiths

**Affiliations:** International Gaming Research Unit, Psychology Division, Nottingham Trent University, Nottingham, UK

**Keywords:** Event frequency, Harm minimisation, Responsible gambling, Self-control, Speed of play

## Abstract

Conceptually, there is a common association between gambling games with fast speeds of play and problem gambling. This relationship however, is largely correlational in nature, which comes at the expense of carefully controlled empirical investigation. Research that does exist aimed towards investigating the impact of gambling speeds on psychological and behavioural factors, is in its relative infancy, and the research possesses disparate methodologies and variables of interest. The aims of the current review is therefore to evaluate and summarise the existing body of evidence relating to speed of play in gambling, as well as discuss how this evidence can be used to inform harm minimisation approaches aimed at facilitating self-control during gambling. Eleven studies were selected for review based on the inclusion criteria, comprising nine experimental and two qualitative studies (one self-report focus group study and one observational study). There was a consistent finding across studies that games with faster speeds of play were preferred and rated as more exciting for all gamblers, ranging from non-problem to problem gamblers. Of concern, was the repeated finding that fast games are particularly appealing to those suffering with a gambling problem. Behavioural results were more inconsistent across studies, though the general trend supports the notion that games with faster speeds of play encourage more wagers, longer game play, and caused players, particularly problem gamblers, to experience difficulty in ceasing gambling. The implications of these findings for gambling policy, harm minimisation approaches, and future research are discussed.

## Introduction

Games with fast speeds of play are frequently associated with problem gambling. For example, it has frequently been observed that problem gamblers seeking intervention or treatment for their disordered gambling often report rapid forms of gambling (such as electronic game machines [EGMs]) as a primary cause of their disordered gambling (e.g., Griffiths 2008; Meyer et al. 2009; Turner and Horbay 2004). In the psychological gambling literature, speed of play is inextricably associated with event frequency, a structural characteristic referring to the number of gambling events within a given time period (and operationalized as the time interval between successive wagers on any given gambling game [Griffiths and Auer 2013]). For example, the event frequency of a bi-weekly lottery is twice a week, whereas the event frequency on an EGM that spins 12 times a minute is five seconds. A fast speed of play has been identified as one of the key features that appeal to gamblers and is therefore more likely to be associated with both higher levels of gambling participation generally, as well as gambling-related harm (Parke and Griffiths 2007). Of concern is evidence suggesting games with fast speeds of play, such as EGMs, are particularly appealing to problem gamblers (Griffiths 2008).

Several theoretical propositions exist that attempt to account for the relationship between high event frequency gambling participation and disordered gambling. For instance, the rapid sequencing of gambling stimuli accompanied with reward (i.e., ‘the constant cycling of player action’; Dow-Schull, 2012) means that that fast, rhythmic, and continuous nature of EGM gambling facilitates an immersive state of lowered conscious awareness for peripheral information. This may give rise to the gambler experiencing a dissociative state, and it has been argued that such psychological states, facilitated by games with fast speeds, are pleasurable to the gambler (Griffiths et al. 2006). During such dissociative experiences, the need for more conscious and deliberate decision-making is limited, providing negative reinforcement to gamble by reducing tension and escaping wider psychological distress that may be experienced in everyday life (Fang and Mowen 2009). However, Norman and Shallice (1986) argue that there are specific situations where the routine activation of behaviour, at the expense of top-down executive control, is maladaptive. Unsurprisingly, among the situations Norman and Shallice (1986) identify include those where potential danger can be experienced, or situations that require planning and decision-making. Given that gambling is a situation requiring the constant updating of goals and adjustment of behaviour, as well as a situation where harm may be experienced, it may be maladaptive for gambling features such as speed of play to facilitate dissociative experiences.

The appeal of games with fast speeds of play, particularly amongst problem gamblers, may also be explained by Gray’s (1970) Reinforcement Sensitivity Theory. The theory postulates that the Behavioural Approach System (BAS) motivates behaviour to seek out reward (Gray 1981, 1991). The subsequent reward, which is exciting and pleasurable to the individual, reinforces the behaviour and consequently leaves individuals highly sensitive to potential rewards and makes extinction of the behaviour difficult. Pickering and Gray (1999) argue that dopaminergic fibres ascending from both the substantia nigra and ventral tegmental areas of the brain, that innervate the basal ganglia, together with motor, sensorimotor, and prefrontal regions, are assumed to drive this system. It has been demonstrated that those with abnormalities in dopaminergic functioning, as well as ventro-medial prefrontal cortex structures, are at risk of developing problem gambling due to abnormalities in the way reward and punishment is processed (Goudriaan et al. 2004). Therefore, it is perhaps unsurprising that gamblers with increased sensitivity to reward will be attracted to games with high event frequencies, as such games are more likely to provide increased levels of reward in a relatively shorter period of time.

Alternatively, sensitivity to punishment or loss is seen as a protective factor in the persistence of risk-taking behaviour (e.g., Gray 1991). Games with high event frequencies also deliver relatively higher rates of loss, and therefore conceptually, one could predict that such factors result in fast games being avoided for gamblers with higher levels of punishment sensitivity. Paradoxically, research demonstrates that this is not the case for gamblers with high levels of sensitivity to reward and punishment. For example, Gaher et al. (2015) argue that the increased sensitivity to punishment results in further gambling to alleviate the negative mood state caused by the loss, which results in loss-chasing behaviours. As a result, reinforcement sensitivity theory is able to predict that those high in either reward sensitivity, and/or punishment sensitivity, would be attracted to and persist on games with fast speeds of play for different reasons.

The rapid and continuous pace of play afforded by gambling games with high event frequencies may potentially interfere with a gamblers’ ability to process new information, update goals, and/or make adjustments in their behaviour to avoid undesirable consequences. Response modulation is a cognitive process whereby the individual disengages attention on the ongoing activity to re-evaluate and adjust behaviour according to the current reinforcement rate of the behaviour in question (e.g., Derevensky et al. 2011). Behavioural perseverance despite negative consequence is a hallmark sign of a wide range of clinical disorders including psychopathy (Newman et al. 1987), borderline personality disorder (Davey 2008), and disordered gambling (Thompson and Corr 2013). Consequently, if a gambler is not afforded the opportunity to pause and reflect between gambling events, it is less likely that they will respond adaptively to punishment (e.g., financial loss). High event frequency games allow less opportunity for such reflection and adaptation of behaviour and are therefore more likely to lead to behaviour symptomatic of problem gambling. In support of this notion, experimental evidence suggests that when problem gamblers are forced to pause for five seconds between events, they do not persist in gambling longer than non-problem gamblers (Corr and Thompson 2014; Thompson and Corr 2013). However, it is unclear whether this effect is due to increased reflection time, or more simply, that the pause made the game less enjoyable. Both factors are not necessarily mutually exclusive.

Whilst these theoretical models have high face validity in explaining why fast speeds of play are associated with disordered gambling, a significant problem remains in that the empirical relationship is largely correlational. The argument can be made that a weak empirical association between fast speeds of play and disordered gambling is potentially harmful to scientific research into this relationship, as it assumes an extensive knowledge-base has already been established. Therefore, one of the goals of the present review is to identify the gaps in the current understanding relating to the impact of high event frequency on gamblers across the entire spectrum of problem gambling behaviour. An additional reason for carrying out the present review paper is to collectively establish what is already known in terms of the psychological and behavioural factors that high event frequency games impact. This is to facilitate the development of gambling harm-minimisation approaches which focus on specific factors that enhance a gamblers’ self-control. As far as the authors are aware, no previous literature review has ever examined speed of play in gambling as the single focus although more general reviews of structural characteristics in gambling have devoted small sections of such overviews to theoretical descriptions of event frequency (e.g., Griffiths 1993; McCormack and Griffiths 2013; Parke and Griffiths 2006, 2007).

## Method

### Search Strategy

An in-depth literature review was carried out comprising three concurrent phases: (i) search of online electronic databases; (ii) use of professional contacts in the field of gambling to share personal collection of papers related to harm-minimisation in gambling; and (iii) ‘snowballing’ - a method in which reference lists from published papers are viewed and relevant papers pursued. Electronic databases included the use of the authors’ Library One Search (an all-encompassing database search engine – including, but not limited to: *Academic Search Elite, PsychArticles, PsychInfo, Science Direct,* and *Scopus*) as a primary source, along with *Google Scholar* being used as a more general search engine. The general search terms used were ‘gambling’, ‘gaming’, ‘electronic gambling’, and ‘online gambling’, with more specific search terms comprising ‘gambling speed of play’, ‘gambling event frequency, ‘responsible gambling’, ‘gambling harm minimisation’, and ‘gambling tempo’.

### Inclusion Criteria

To be included as an output to be evaluated, the published paper had to have: (i) been written in the English language; (ii) reported a study where speed of play was an independent/dependent variable, a predictor/outcome variable, or an area of interest for qualitative studies (e.g., observational studies, interview studies, etc.); (iii) been published within the last 25 years (1991–2016); and (iv) been subjected to peer-review. It was assumed that those studies that had undergone peer-review would be more scientifically rigorous than anything in the ‘grey’ literature.

### Search Results

Once the initially retrieved papers had been filtered according to title and abstract content, a more in-depth assessment was conducted using the inclusion criteria as guidance. The remaining papers were then categorised according to the type of study reported: experimental or qualitative. Using this method, a total of 11 studies remained for critical review comprising nine experimental studies and two qualitative studies (one focus group interview study and one observational study). A summary of the reviewed papers can be found in Table 1. The studies are critically reviewed in chronological order.

**Table 1:** Summary of research papers (n = 11) selected for review and the design, main aims and main findings

Author(s) (Year)	Country	Main aims	Sample (N) (Design/method)	Key finding(s)
*Experimental research studies*				
Griffiths (1994)	United Kingdom	To assess the cognitive biases demonstrated by regular non-problem gamblers and non-regular non-problem gamblers. Speed of play was one of the dependent variables	30 regular non-problem gamblers (29 males) and 30 non-regular non-problem gamblers (15 male). Mean age = 23.4 years. (Experiment in a real gambling venue)	Regular gamblers gambled significantly more times per minute (n = 8) compared to non-regular gamblers (n = 6)
Loba et al. (2001)	Canada	To determine which gambling structural manipulations, including speed of play, might help reduce the risk of abuse of VLTs by pathological gamblers	60 regular VLT players (38 males), with 29 being classed as a pathological gambler and 31 as non-pathological gamblers, as determined by the SOGS. Mean age = 34.7 years (SD = 11.6) (Laboratory-based experiment using commercially available VLT)	Compared to non-pathological gamblers, pathological gamblers’ ratings of enjoyment, excitement, and tension-reduction was significantly reduced when speeds of play were reduced, as well as when sound was turned off during the game. Pathological gamblers reported significantly more difficulty in stopping gambling than non-pathological gamblers when speed of play was increased accompanied by sound
Blaszczysnki et al. (2005)	Australia	To investigate the impact of structural manipulations, including speed of play, on subjective gambling experience in a live gambling setting	400 participants of various non-problem and problem gambling statuses (Naturalistic EGM experiment in real gambling venues)	Satisfaction ratings were reduced significantly when both social and problem gamblers played the machines modified to produce a 5-s event frequency compared to 3-s event frequency. There was a non-significant impact of slowing the event frequencies on self-reported enjoyment levels
Delfabbro et al. (2005)	Australia	To investigate the impact of parameter variation, including speed of play on a simulated EGM, in terms of their impact on subjective gambling experience and observable gambling behaviour	24 gamblers with various gambling experience (15 males), participation rates, and problem gambling statuses. Mean age of participants in Experiment 3 was 47.92 years (SD = 15.6), with 10 of the gamblers being classed as a problem gambler using the SOGS. (Laboratory-based experiment using simulated EGM)	Faster speeds of play (3.5-s event frequency) yielded a significantly higher excitement rating than slower speed games (5-second event frequency). Preference ratings were significantly higher for faster speed machines. No significant impact of speed of play on the amount spent gambling, but the total amount of games played was significantly higher in the faster speed condition
Sharpe et al. (2005)	Australia	To investigate the impact of structural manipulations, including speed of play, on gambling behaviour in a live gambling setting	779 gamblers, from which, 634 participants provided SOGS scores. One-fifth (20%) of the sample were classed as problem gamblers having scored five or more on the SOGS. All other participants were grouped as non-problem gamblers. Participant mean age was 46.1 years (SD = 17.9) years (Naturalistic EGM experiment in real gambling venue)	The speed manipulations (3.5, 5 s) had little effect on gambling behaviour. There was no statistical significance in terms of the difference in time spent on the gaming machines, number of bets placed, amount of money lost, number of lines or credits played, and alcohol and cigarette consumption, as a result of manipulations in speed of play
Ladouceur and Sevigny (2006)	Canada	To investigate the impact of VLT speed on gamblers’ levels of concentration, motivation, self-control, and the amount of games played	43 regular and non-regular non-problem gamblers (22 females). (Laboratory-based VLT simulation experiment)	Gamblers in the 5 s condition played more games and underestimated the number of games they had played compared to participants in the slow (15 s) speed condition. Speed of play did not however, have a statistically significant impact on participant levels of concentration during gambling, motivation to continue gambling, or time and money spent gambling
Linnet et al. (2010)	Denmark	To investigate the effects of event frequency on the behaviour and experiences of problem and non-problem gamblers	15 pathological gamblers (10 males) and 15 non-problem gamblers (8 males). (Laboratory-based experiment using a commercially available VLT)	Pathological gamblers reported significantly higher levels of excitement in the fast (2-s) condition compared to non-problem gamblers. This significant effect was not maintained in the slower (3-s) condition. Pathological gamblers also reported significantly higher desire to play again than non-problem gamblers in the 2 s condition. Pathological gamblers spent more time gambling than non-pathological gamblers in both the 2-second and 3-second condition. Significantly more pathological gamblers (60%) continued gambling until they were told to stop in the 2-second condition compared to non-pathological gamblers (6.7%)
Choliz (2010)	Spain	To investigate impact of different reward delays, and thus, event frequency, on gambling behaviour among treatment seeking problem gamblers	10 treatment seeking problem gamblers. (Laboratory-based experiment using a simulated slot machine)	More games were played in the 2-second (immediate reward) event frequency condition (n = 56) when compared to the 10-second (delayed reward) condition (n = 39)
Mentzoni et al. (2012)	Norway	To investigate the impact of various bet-to-outcome-intervals (BOI; and thus, speed of play) on subjective gambling experience, illusions of control, and observable gambling behaviour	62 undergraduate students (31 males) with a mean age of 20.8 years (SD = 3.26). Three participants were probable pathological gamblers, 27 had some problems with gambling, and 32 had no problems with gambling (using SOGS) (Laboratory-based experiment using computer simulated slot machine)	No overall main effect of BOI on average bet size, illusion of control, or subjective enjoyment ratings, and no evidence that the faster game was preferred by the participants. However, results, indicated an interaction effect, at-risk pathological gamblers made significantly higher average bet sizes than non-problem gamblers in the short (fast speed) BOI condition
*Qualitative research studies*				
Griffiths (1999)	United Kingdom	To observe amusement arcade clientele and their behavioural characteristics	Hundreds of adolescent gamblers in 33 UK-based amusement arcades across various parts of England. (Observational field study)	Common amongst regular gamblers was that they played at very fast speeds of up to 100 times in 10 min. Fast-paced gamblers appeared to be on ‘automatic pilot’, a state which was only halted temporarily when the ‘nudge’ feature of the slot machine came into play
Thompson et al. (2009)	United Kingdom	To enhance understanding of how structural characteristics of gaming machines interact with the gambler	48 gamblers, with statuses ranging from non-problem to current problem gambler. (Series of interviews and focus groups)	Speed of play was identified as a core structural characteristic that drives gambling behaviour, and faster games reported to enhance the gambling experience

## Results

### Experimental Studies

Griffiths (1994) conducted an ecologically valid gambling experiment in the UK using slot machines in a gambling arcade to assess both the cognitive biases by regular non-problem gamblers (N = 30, 29 males) and non-regular non-problem gamblers (N = 30, 15 males), as well as their overt gambling behaviour (mean age = 23.4 years). Cognitive biases were assessed using the ‘thinking aloud’ method where gamblers’ verbalisations are recorded and categorised (Ericcsson and Simon 1980). Overt gambling behaviour variables included total plays, time spent gambling, and speed of play. Results relating to speed of play demonstrated that on average, regular gamblers played significantly faster (eight gambles per minute) compared to non-regular gamblers (six gambles per minute). The mean speed of play rate was reduced in the thinking out loud condition for non-regular gamblers from 6.5 to 5.3 gambles per minute, and increased in the thinking out loud condition for regular gamblers from 7.5 to 8.4 gambles per minute, though both of these differences were not statistically significant.

Because cognitive biases were the main focus of this experiment and not speed of play, and the fact that speed of play was used as one of several dependent variables, knowledge gained in terms of the impact of speed on the gambler is limited. However, the study did provide empirical evidence that regular gamblers play on slot machines significantly quicker than non-regular gamblers (*p* < .01). Reasons for this may simply be due to the fact that regular gamblers are more familiar with the gambling product and consequently, the game mechanics, allowing them to operate the games at a faster pace through familiarity and competence. This was supported by the verbalisations from both regular and non-regular gamblers in the ‘thinking aloud’ condition. Compared to regular gamblers, non-regular gamblers made significantly more verbalisations that were classed as ‘confused questions’ (*p* < .001) and ‘confused statements’ (*p* < .001), suggesting that the lower level of competence may slow down the speed of gambling for non-regular gamblers.

Loba et al. (2001) conducted a laboratory-based experiment in Canada using commercially available video lottery terminals (VLTs) to examine the effects of structural characteristic manipulations on subjective game experiences. Participants comprised 60 regular VLT players (38 males), with 29 being classed as a ‘pathological gambler’ and 31 as ‘non-pathological’ gamblers, as determined by the SOGS (Lesieur and Blume 1987). Participants were on average 34.7 years of age (SD = 11.6). Game manipulations included increasing and decreasing the speed of play for a video poker and ‘reel spin’ game, as well as other sensory manipulations such as sound/no sound, stop button/no stop button, and display counter/no display counter. Results indicated that when compared to non-pathological gamblers, pathological gamblers’ ratings of enjoyment, excitement, and tension-reduction was significantly reduced when speeds of play were reduced, as well as when sound was turned off during the game. Of note, pathological gamblers reported significantly more difficulty in stopping gambling than non-pathological gamblers when speed of play was increased accompanied by sound.

However, it is not made clear to what extent the game speeds were increased or decreased relative to a control condition, as no information on VLT event frequency was provided. This is an important omission, as it is not known if the pathological gamblers were sensitive to small changes in event frequency, or if in fact the speed manipulations were large. In addition, the use of dichotomous participant groupings, non-pathological vs pathological gamblers, overlooked the fact that pathological gambling behaviour is viewed along a continuum of problematic behaviours and intensities, where several intermediate levels of risk between non-pathological and pathological gambling exist (Currie and Casey 2007). In terms of the impact of speed of play on self-reported gambling experiences, it is important to acknowledge that speed of play was manipulated concurrently to other multiple structural game changes. This makes it difficult to ascertain the proportional impact of each manipulation on reported gambling experiences, and therefore does not shed light on the impact of speed of play on gambling experiences in isolation. However, it is understandable why speed was not isolated in Loba et al.’s experimental procedure given the already lengthy experiment duration (i.e., two hours).

Sharpe et al. (2005) conducted a naturalistic experiment, in which various structural manipulations to eight gaming machines in gambling venues and hotels in the New South Wales region of Australia were made. Participants comprised 779 gamblers, from which 634 participants provided SOGS scores. Participant mean age was 46.1 years (SD = 17.9), and the mean SOGS score was 2.43 (SD = 3.43) out of 20. One-fifth (20%) of the participants were classed as problem gamblers having scored five or more on the SOGS. All other participants were grouped as non-problem gamblers due to sub-categories of ‘at-risk’ gamblers being too small for reliable statistical analysis. Speed of play was one of the independent variables, being manipulated at two levels: 3.5-second, and 5-second event frequencies, with maximum bet size and maximum size note acceptors as the two other structural characteristics being experimentally manipulated.

The speed manipulations had little effect on gambling behaviour. There was no statistical significance in terms of the difference in time spent on the gaming machines, number of bets placed, amount lost, number of lines or credits played, and alcohol/cigarette consumption, as a result of manipulations in speed of play. However, it is not possible to tell from this study whether reductions in speed of play would be differentially effective for problem gamblers as compared to non-problem gamblers, because there were insufficient numbers of problem gamblers included in the study. In addition, that fact that gambling behaviour was being observed by the researchers may in turn have produced demand characteristics, possibly resulting in gamblers behaving in a more controlled and moderate manner, gambling more slowly and deliberately as a result. Of the three proposed modifications, only a reduction in maximum bet size to $1 demonstrated evidence for a potential reduction in harm associated with gambling, because those gambling on $1 maximum machines played for less time, made fewer bets, lost less money, and consumed less alcohol and cigarettes during play.

Blaszczynski et al. (2005) similarly demonstrated that a reduction in speed of play on EGMs from a three-second to five-second event frequency had no impact on a gambler’s intentions to continue playing. They conducted a live experiment in hotels and clubs in the Sydney region of Australia, comprising more than 400 participants of various non-problem and problem gambling statuses who played on modified experimental and non-modified gaming machines. As well as manipulating speed of play, experimental machines were modified to limit the maximum bet size and reduce the high denomination note acceptors compared to control machines. Limiting the maximum bet size and note acceptor modifications had a non-significant impact on self-reported satisfaction and enjoyment levels for both social and problem gamblers. However, satisfaction ratings were reduced significantly when both social and problem gamblers played the machines modified to a five-second event frequency, when compared to the unmodified machines with three-second event frequencies. There was a non-significant impact of slowing the event frequencies on self-reported enjoyment levels, although Blaszczynski et al. (2005) report this as a trend towards reduced enjoyment levels given the *p* value of .065. There was no interaction effect between levels of enjoyment of three- and five-second event frequencies and problem gambling status, although overall, problem gamblers rated all EGMs as less enjoyable than social gamblers. While satisfaction ratings reached statistical significance, the largest difference in satisfaction and enjoyment scores between the modified and control machines was just 8.75%, suggesting a small effect size.

Despite the seemingly negative impact of reducing speed of play on satisfaction and enjoyment levels, this did not impact gamblers’ intentions to continue gambling on EGMs, as respectively, 54% and 53% reported intentions to continue play on the control and experimental machines. Speed of play was the only modification to the machines that gamblers were able to identify, although detection rates were low, with only 14% of gamblers able to identify the modifications. This suggests that reasons for the reduced satisfaction and enjoyment ratings were subconscious, at least for the majority of the gamblers in this experiment. An alternate explanation could be that the overall effect of reduced satisfaction and enjoyment was driven only by those gamblers that were able to detect the reduced speed modification. Further post hoc statistical analysis would be required to provide evidence for such claims.

Delfabbro, Falzon and Ingram (2005) conducted three laboratory-based experiments in South Australia assessing the impact of parameter variation on simulated EGMs in terms of their impact on subjective gambling experience and observable gambling behaviour. The EGM manipulations included reinforcement magnitude and frequency (Experiment 1), sound and screen illumination (Experiment 2), and outcome display and speed manipulation (Experiment 3). The speed of play in Experiment 3 was manipulated at two levels to provide machines with both a 3.5- and five-second event frequency.

Participants exposed to the speed of play manipulations were 24 gamblers (15 males) with various gambling experiences, participation rates, and problem gambling statuses. The mean age of participants in Experiment 3 was 47.92 years (SD = 15.6), with 10 of the gamblers being classed as a problem gambler using the SOGS. Participants were asked to play for three minutes on each of the four machines programmed with the varying parameter settings (credit display/fast speed, credit display slow speed, dollar display fast, and dollar display slow). After this mandatory exposure, participants were given a free choice to continue gambling on one of the four machines.

Speed of play was shown to significantly influence excitement ratings, with faster speeds yielding a significantly higher rating than slower speed games. Preference ratings were again, significantly higher for faster speed machines. Display type (dollars vs. credits) did not significantly impact excitement or preference ratings. There was no significant impact of speed of play in terms of the amount spent gambling on the machines overall, but the total amount of games played was significantly higher in the fast speed condition. Control measures indicated that these differences in subjective experience ratings and gambling behaviour could not simply be attributed to specific machines yielding a higher return to player or win rate, indicating the effects were driven by the speed manipulations alone. Neither gender, nor problem gambling status, interacted with the manipulations to produce significant effects, though these small sub-sample comparisons may not be reliable given the low number of participants in each category (e.g., Experiment 3 comprised just 10 problem gamblers).

Ladouceur and Sevigny (2006) investigated the impact of VLT game speed on gamblers’ levels of concentration, motivation, self-control, and number of games played. Participants comprised 43 gamblers (22 females) from the Quebec City region of Canada. Gambling participation rates ranged from 0-24 times over the past six months, with an approximate overall mean average of three times in the past six months. A majority of the sample (n = 32) scored zero on the SOGS, six had a score of one, and five had a score of two, indicating the sample did not contain any problem or at-risk gamblers.

Speed of play was manipulated at two levels, with one group being exposed to a VLT game with a five-second event frequency, the other group a 15-second event frequency. Gamblers in the five-second condition played more games and underestimated the number of games they had played compared to participants in the slow speed condition. However, speed of play did not have a statistically significant impact on participant levels of concentration during gambling, motivation to continue gambling, or self-control in terms of time and money spent gambling. The authors concluded that the slower speed VLT game did not appear to have any positive impact in terms of facilitating more controlled gambling behaviour among the participants studied.

The use of both a five-second event frequency for the ‘fast’ condition and 15-second event frequency for the ‘slow’ condition is questionable, particularly given that event frequencies on electronic gaming machines can reach three seconds for offline EGMs, and even higher ones in their online form. Consequently, a five-second event frequency would arguably be considered slow for specific forms of EGM gambling. Motivation to continue playing was extremely low in both speed conditions, with mean motivation scores of 2.6 and 2.5 out of 10 being recorded in the fast and slow conditions respectively. Enjoyment ratings of both games were also arguably very modest, with mean enjoyment ratings 2.7 and 2.5 out of 4 for the fast and slow condition respectively. Of note, was that 67% of participants in the slow condition reported that they would like the game to go faster (compared to just 33% in the fast condition). Taken together, it could be argued that the gambling in this experimental study failed to replicate the exciting and arousing nature of real-world gambling, although it is acknowledged that this is often a trade-off for high-levels of experimental control. In addition, mean participation rates in gambling were very low for this sample, with mean participation rates equating to just once every couple of months, meaning that the gamblers were already participating at highly controlled levels, potentially masking the effects of the speed modification, and failing to be representative of gambling behaviour typically exhibited by more regular gamblers.

Linnet et al. (2010) conducted a laboratory-based experiment in Denmark to investigate the effects of event frequency on the behaviour and experiences of problem and non-problem gamblers. The study comprised 15 pathological gamblers (10 males) and 15 non-problem gamblers (eight males). Event frequency on a popular and commercially available slot machine was manipulated at two levels to produce a two-second and three-second event frequency slot machine. The dependent variables included self-reported excitement levels, desire to play again, and time spent gambling.

Pathological gamblers reported significantly higher levels of excitement in the two-second condition compared to non-problem gamblers. This significant effect was not maintained in the three-second condition. Pathological gamblers also reported significantly higher desire to play again than non-problem gamblers in the two-second condition, but again, this effect was not maintained in the three-second condition. Pathological gamblers spent more time gambling than non-pathological gamblers in both the two-second and three-second condition. In addition, significantly more pathological gamblers (60%) continued gambling until they were told to stop in the two-second condition compared to non-pathological gamblers (6.7%). In the three-second condition, twice as many pathological gamblers (40%) continued gambling until stopped compared to non-pathological gamblers (20%), although this effect did not remain statistically significant.

Overall, the results supported the notion that the behaviour and gambling experiences of pathological gamblers differs significantly from non-pathological gamblers at the faster two-second event frequency, but that their behaviour and experience was more similar at the slower three-second event frequency. However, upon close examination of the statistics, pathological gamblers report approximately 40-60% higher ratings of excitement and desire to continue gambling compared to non-pathological gamblers in the three-second condition. While these figures did not differ at a statistically significant level, this lack of statistical significance is likely due to the small sample size of just 15 for each problem gambling status, and represents a significant limitation of the study. An additional limitation of the experimental procedure was that the experimenters were not able to control payback and win percentages across the two slot machines. As a result, the researchers were not able to control for extraneous variables such as emotion as a result of wins and losses, which has been demonstrated to be an important determinant in a range of gambling behaviours (Harris and Parke 2015; Harris et al. 2016).

Choliz (2010) manipulated reward delay, and thus, event frequency in a repeated-measures laboratory experiment conducted in Spain. The sample comprised 10 problem gamblers recruited from gambling treatment services, and they took part in a simulated slot machine study. Whilst controlling for reel speed, the experimenter manipulated the reward delay at two levels: a two-second, and 10-second delay. While the reward delay was the main variable of interest, it is important to note that as a result of this experimental manipulation, event frequency duration coincided with the reward delay, to produce a condition with a two-second and 10-second event frequency.

Key results indicated that more games were played in the two-second event frequency condition (n = 56) when compared to the 10-second condition (n = 39). Choliz (2010) reported that this difference could not be attributed to volume or frequency of winning outcomes because there were no significant differences in gambling outcome across the two conditions. However, it is questionable whether results were driven by the reward delay or the event frequency. It may have been the case that fewer games were played in the ten-second condition due to each game cycle taking longer to complete, and participants may simply be constrained for time resulting in fewer games being played. Caution must also be taken relating to the reliability of results given the small sample of just 10 participants.

In a Norwegian laboratory-based experiment using a computer-simulated slot machine, Mentzoni et al. (2012) investigated the impact of various bet-to-outcome-intervals (BOIs) on subjective gambling experience, illusions of control, and observable gambling behaviour. The authors define BOI as the time delay between the initiation of the bet and receiving the outcome of that bet. However, there was an important distinction overlooked by Mentzoni et al. (2012) between event frequency and BOI. It is possible to have a short BOI structure within a relatively slower event frequency if the outcome of the event does not signify the point at which a new game cycle or bet can begin. For example, a slot machine may spin for two-seconds and reveal the outcome of the wager immediately following the reel spin, but there may be a further one-second delay before a new wager can be made. In this hypothetical example, the machine would have a two-second BOI, yet a three-second event frequency. This distinction is not made by the authors, so it has to be assumed that BOI and event frequency are of the same length of time in this study.

Sixty-two undergraduate students (31 males) with a mean age of 20.8 years (SD = 3.26) participated in the between-participants experiment. Three participants were probable pathological gamblers, 27 had some problems with gambling, and 32 had no problems with gambling, as indicated by the SOGS. Of note, the three participants scoring five or more on the SOGS were excluded from further analysis. Participants were allocated to one-of-three BOI condition: 400 ms; 1700 ms; and 3000 ms respectively. The results showed no overall main effect of BOI on average bet size, illusion of control, or subjective enjoyment ratings, and therefore, little evidence to support the notion that speed of play leads to more intensive and risky gambling, and no evidence that the faster game was preferred by the participants. However, results did indicate an interaction effect in that at-risk pathological gamblers made significantly higher average bet sizes than non-problem gamblers in the short BOI condition. The differences in bet sizes between these two sub-groups did not reach statistical significance in the moderate or long BOI condition. This may indicate that at-risk gamblers may be particularly susceptible to elevated risk-taking in games with high event frequencies and short BOIs.

To reiterate, one of the limitations of this study was the lack of distinction between BOI and event frequency, so it is not possible to ascertain whether the short BOI or high event frequency resulted in at-risk gamblers escalating their average bet sizes. Further research would be required to control for this distinction. In addition, the absence of a meaningful sample size of pathological gamblers means results cannot be extended to account for the behaviour of those at the extreme end of the problem gambling continuum.

### Qualitative Studies

Thompson, Hollings and Griffiths (2009) conducted a qualitative investigation into EGM gambling, with one of their key objectives being to gain an enhanced understanding into how structural characteristics of machines interact with the gambler. Forty-eight gamblers, with statuses ranging from non-problem to current problem gambler, participated in a series of interviews and focus groups across several regions of the UK. Throughout the investigation, speed of play was identified as a core structural characteristic that drives gambling behaviour. The instantaneous nature of machine play, and the real-time risk involved was found to be a key motivation for many players. These factors were enhanced by the speed of machine gambling compared to some of the other forms of gambling. Two of the recovering problem gamblers stated how they preferred electronic roulette in its’ virtual form (played via ‘fixed odds betting terminal’ machines) because less time is wasted counting and raking chips compared to ‘live’ roulette:

“I played roulette on the table and it wasn’t quick enough for me. I was too impatient, I couldn’t wait. So I’d play the machines” (Problem gambler).

“They’re very fast. A gambler’s trait is impatience and there’s no waiting around … It’s just you and the machine, pressing the button” (Problem gambler).

The authors highlighted how several problem gamblers likened rapid machine play to taking drugs:

“I like the instant fix, the constant fix” (Problem gambler).

Several gamblers reported using the ‘autoplay’ feature to facilitate faster play. Other ways a minority of gamblers reported trying to increase the game speed was by playing multiple machines simultaneously or betting on multiple lines. Findings demonstrated that the majority of problem gamblers reported a preference for simpler games, such as three-reeled slots, with no bonus boards, as it was these simple machine variants that allowed for faster rates of play and thus, more opportunities to win. A smaller proportion of problem gamblers along with regular gamblers reported that they preferred slower and more complex games with a larger skill element. These were the players who reported gambling to kill time. Additionally, the slower pace and increased complexity allowed for longer periods of gambling:

“You’ve got to do more so it makes your money last longer” (Regular gambler).

From this qualitative study, it appears that there is a tendency for those with elevated levels of problem gambling to prefer games of a rapid nature to maximise excitement and wins. However, regular non-problem gamblers had a tendency to report a preference for more complex and slower games to allow them to play for longer. The authors’ reported the particularly fruitful nature of the one-to-one interviews where problem gamblers were able to disclose more personal and experiential information in a confidential manner. This was not the case in the focus groups, therefore, this part of the study may have suffered from well-reported limitations of focus group research in that those with more dominant personality and communication may have overrepresented the views of the majority.

In a different type of qualitative study, Griffiths (1999) conducted a longitudinal observational study across 33 inland and coastal amusement arcades over a 28-month period. Although no specific hypotheses were made because of the exploratory nature of the observations, one of the general aims of the research was to observe amusement arcade clientele and their behavioural characteristics. Relating to the present aims of this review, Griffiths observed that a commonality amongst regular gamblers was that they played at very fast speeds of up to 100 times in 10 min. The study described these fast-paced gamblers as being on ‘automatic pilot’, a state which was only halted temporarily when the ‘nudge’ feature of the slot machine came into play. These observations suggest an altered state of conscious awareness and narrowing of attention was produced for regular gamblers playing EGMs at fast speeds. The findings also suggest that specific game characteristics such as ‘nudge’ features have the potential to break lowered conscious and autopilot states.

However, Griffiths (1999) did not define regular gamblers (except that he recognised regular patrons over the course of the longitudinal study), and given the non-intrusive observational nature of the research, it was not possible to obtain the players’ problem gambling status. As a result, it is difficult to ascertain if the rapid pace of play observed in regular gamblers was a result of any underlying gambling problems, or the structural features of the games themselves inducing a rapid play style. Of note, while it was observed that specific game features (i.e., nudges) appeared to break dissociative states, it might be the case the benefits of this are offset by the increased illusion of control, which has shown to be a predictor of problem gambling behaviour (Fu and Yu 2015).

## Discussion

Based on the studies reviewed, there appears to be an overall trend from the experimental findings that games with high event frequencies are perceived as more exciting and more enjoyable by gamblers, and which is likely to be one of the core factors accounting for the popularity of EGMs. This is a finding that applies to gamblers across the entire problem gambling continuum. This evidence is supported and complemented by the qualitative data surrounding speed of play, where the reasons gamblers show a preference for such fast games include the instant gratification they provide, and the lack of waiting around between gambling events which appeals to the gamblers’ ‘lack of patience’. However, while both problem and non-problem gamblers rate faster games as more enjoyable when compared to slower game speeds, some studies (e.g., Linnet et al. 2010) demonstrate that enjoyment ratings for fast games are significantly higher amongst problem gamblers. Furthermore, some studies found that problem gamblers also report a significantly higher desire to continue gambling on faster games when compared to the same ratings made by non-problem gamblers, as well as problem gamblers also reporting a greater reduction in tension when playing faster games. Taken together, these findings appear to support previous notions that games with fast speeds are particularly appealing to those displaying signs of disordered gambling (e.g., Griffiths 2008).

In terms of the behavioural impact of speed of play, results demonstrated a varied set of findings. Several studies reported that games with faster speeds of play resulted in more games being played compared to slower games (e.g., Loba et al. 2001; Delfabbro et al. 2005; Ladouceur and Sevigny, 2006), which is perhaps unsurprising given the fact that a higher event frequency affords the gambler the opportunity to make more bets in a given period of time compared to games with slower event frequencies. Several studies also found that problem gamblers reported more difficulty in stopping gambling compared to non-problem gamblers at fast speeds of play (an effect that disappeared when game speed was slowed) or that problem gamblers were significantly more likely to continue gambling until asked to stop at fast speeds compared to non-problem gamblers (an effect that was also found at slower speeds, though to a lesser extent). One study (i.e., Mentzoni et al. 2012) showed that speed interacted with problem gambling status, demonstrating that problem gamblers significantly increased their average bet sizes in games with fast speeds. Taken together, these results suggest problem gamblers have more difficulty in exercising self-control compared to non-problem gamblers regardless of speed of play, but that this effect is exacerbated with fast game speeds. However, several studies showed that speed of play had no impact on variables including both the amount of time and money spent gambling, number of bets placed, desire to continue gambling, and illusion of control. The trend appears to point towards an overall deleterious impact of speed of play on gambling behaviour and self-control, but results are inconsistent. This inconsistency is most likely due to the relatively small amount of studies conducted investigating speed of play, the varied methodologies used among this small sample, and the methodological limitations they possess (particularly the relatively small sample sizes). Coupled with this, the present review clearly demonstrates that there is a lack of studies with longitudinal designs and that those studies with small sample sizes include relatively few individuals with gambling problems making it difficult to provide any definitive conclusions regarding the impact of speed of play on both problem and non-problem gamblers and/or the differences between them.

The examination of the impact of speed of play on gambling behaviour in a real gambling venue, using commercially available gambling products, has the advantage of assessing gambling behaviour in its’ natural environment. While this adds a great deal of ecological validity to the findings, a drawback is that tight experimental control measures are sacrificed. For example, it has been found that the structural changes made to machines in such studies are often not made in isolation. That is to say, speed of play was not the only variable manipulated, making it more difficult to ascertain casual influence on gambling behaviour. Of note, several of the laboratory-based studies also fall victim to this limitation, but as a whole, experimental research in the laboratory environment has the added advantage of implementing higher levels of experimental control and more easily isolating the impact of speed of play on gambling behaviour. Whilst it is acknowledged that gambling comprises a wide range of structural and situational characteristics (McCormack and Griffiths 2013; Parke and Griffiths 2007), a more parsimonious approach is essential to learn more about specific structural characteristics (in this case speed of play) and its’ influence on the gambler.

Another factor potentially driving the inconsistent findings is the nature of the speed of play manipulation in this body of research. As a case example, the ‘fast’ speed of play condition in Ladouceur and Sevigny’s (2006) experimental study consisted of a five-second event frequency, whereas this would not even qualify as the slow condition in both Mentzoni et al.’s. (2012) and Linnet et al.’s. (2010) experiments, and only matched the speed of the slow condition in Delfabbro, Falzon, and Ingram’s (2005) experiment. This has important implications in the way results are interpreted and reported. It may be more beneficial for research findings to be interpreted in terms of the event frequency itself (measured in seconds, for example), rather than any subjective interpretation of what makes for a ‘fast’ or ‘slow’ condition. This way, results can be standardised and made to be more comparable across studies, and also has the added advantage of helping to ascertain speed thresholds where self-control becomes facilitated or degraded. It would also be advisable for speed manipulations to be anchored and manipulated proportionally around industry standard event frequencies, which occur approximately every three seconds on EGMs, allowing event frequency speed results to be assessed against existing industry benchmarks. Furthermore, there are relatively few studies that manipulate speed comparable to the faster pace of games found on online gambling platforms, emphasising the need for investigations evaluating the impact of both decreases and increases in speed of play.

There is also the argument that the impact of speed of play may not be immediately visible by assessing direct and overt gambling behaviour in some cases. The impact may be more subtle, and not captured within a relatively short experimental session, where the effects of speed on behaviour may take impact over a more sustained period of time by influencing executive functions vital for self-control not assessed in these studies. For example, these studies did not assess core executive functions such as response inhibition, reflection impulsivity, or response modulation, functions which act as the antithesis to a more impulsive style of response, and functions that act as predictors of risk-taking behaviours (Mahmood et al. 2013). Emerging evidence has demonstrated that executive control capabilities can be influenced by structural characteristics in a gambling context, characteristics that include stake size (Parke et al. 2015), as well as speed of play (Harris and Griffiths 2016). Furthermore, evidence suggests that facilitating response inhibition in a gambling context leads to a preference for less risky gambling-related decisions (Verbruggen et al. 2012).

The present review identified just one study utilising a qualitative self-report approach, the findings from which supported the empirical studies in which gamblers frequently report a preference and increased levels of enjoyment in games with high event frequencies. Obtaining first-person perspectives may offer fruitful information not otherwise available to gambling harm-minimisation research studies via the experimental method. Interviews and focus groups may provide insights into alternative ways of facilitating self-control during gambling, without excessively slowing down the speed of the game, which has been shown to have a consistent detrimental impact on the enjoyment of gambling. For example, gamblers report the need for ‘a constant fix’, so one avenue of exploration may be to find ways of providing breaks in play to facilitate self-control and allow for response modulation, but whist simultaneously making the time between gambling entertaining, such as the use of non-gambling mini-games. It is also advisable that gamblers are involved in the design process of such measures, much the same way that gambling focus groups were used to help create persuasive system designs to facilitate monetary limit adherence tools in a study conducted by Wohl et al. (2014).

## Conclusions

Despite much reference to problem gambling being associated with games with high event frequencies (e.g., EGMs), research actually investigating the impact of speed of play on gamblers is in its relative infancy. The majority of the limited empirical evidence points towards the notion that games that have a faster speed of play are more enjoyable and desirable by an array of gamblers, but that this comes at the cost of impaired self-control. The increased number of bets placed, increased time spent gambling, and the reduced ability or willingness to stop gambling during fast games, appears particularly applicable to, but not limited to, problem gamblers, suggesting close attention should be paid towards implementing measures to facilitate self-control during rapid forms of gambling. Slowing down game speed has shown some (but inconsistent) support for reducing risk-taking and facilitating self-control, although evidence suggests this would likely reduce gambling enjoyment and detract from the experience of gambling. As a result, potential perverse and unintended consequences may result from slowing game speeds, in the form of compensatory behaviours or a migration to other products. For example, if game speed is slowed, this may result in gamblers making higher volume bets to compensate for the reduced event frequency, or playing multiple products simultaneously to essentially allow for the same volume of gambling in the same period of time. Alternatively, slowing game speed on EGMs may result in gamblers migrating to online forms of gambling, where speeds of play can be much faster, and where the online environment itself can give rise to increased risk-taking and reduced inhibitions (Suler 2004).

As an alternative, researchers should investigate ways of implementing harm-minimisation tools that have the effect of making gambling safer by facilitating self-control, but that are less conducive to detracting from the overall enjoyment and experience of gambling such as slowing game speeds. One possibility mentioned is the use of non-gambling mini-games during breaks in play to both provide a chance to take a break and modulate behaviour, but maintain entertainment levels. Existing research also suggests that while breaks in play in isolation may be detrimental to gambling behaviour by increasing cravings and negative subjecting emotion (Blaszczynski et al. 2016), when breaks are accompanied by responsible gambling messages that are not overly paternalistic and allow gamblers to engage in self-appraisal (Monaghan and Blaszczynski 2010), or allow a gambler to focus on external emotional factors (Harris et al. 2016), gambling behaviour is shaped more positively.

Therefore, rather than slow down the speed of the game (which would likely decrease the pleasure of gambling for those without any problems), gambling operators should utilize gambling tools that promote responsible gambling (Harris and Griffiths 2016). There is now growing empirical evidence that some responsible gambling tools can help decrease the time and money spent playing among individuals who gamble intensely on games with fast speeds of play including pre-commitment tools such as limit-setting features (Auer and Griffiths 2013) and personalised feedback based on actual gambling behaviour (Auer and Griffiths 2015, 2016). Unfortunately, such tools can only be used on those gambling games where playing behaviour can be electronically tracked such as those online and/or those that require a loyalty card or player card to gamble. However, some operators in some countries (such as *Norsk Tipping* in Norway and *Svenske Spel* in Sweden) use mandatory player cards that tracks all gambling behaviour both online and offline and such a system could be implemented by other operators in other countries.

Finally, further research is required to ascertain the psychological mechanisms that mediate the relationship between speed of play and overt gambling behaviour. It is possible that the total impact of high event frequencies on the gambler is not immediately captured within short, single-session experimental procedures (which is why, as mentioned above, longitudinal research is needed), or that it is not immediately observable using overt gambling behaviour. What may be required is the use of proxy measures deemed essential for the application of self-control, particularly relevant in a gambling context. Such measures may include core executive functions that act as the antithesis to impulsivity, for example response inhibition and response modulation.
